# The X-Ray Transform Projection of 3D Mother Wavelet Function

**DOI:** 10.1155/2013/754829

**Published:** 2013-11-27

**Authors:** Xiangyu Yang, Jiqiang Guo, Li Lu, Li Zeng

**Affiliations:** ^1^College of Mathematics and Econometrics, Hunan University, Hunan 410082, China; ^2^ICT Research Center, Key Laboratory of Optoelectronic Technology and System of the Education Ministry of China, Chongqing University, Chongqing 400044, China; ^3^College of Mathematics and Statistics, Chongqing University, Chongqing 401331, China

## Abstract

As we all know, any practical computed tomography (CT) projection data more or less contains noises. Hence, it will be inconvenient for the postprocessing of a reconstructed 3D image even when the noise in the projection data is white. The reason is that the noise in the reconstructed image may be nonwhite. X-ray transform can be applied to the three dimensional (3D) CT, depicting the relationship between material density and ray projection. In this paper, nontensor product relationship between the two dimensional (2D) mother wavelet and 3D mother wavelet is obtained by taking X-ray transform projection of 3D mother wavelet. We proved that the projection of the 3D mother wavelet is a 2D mother wavelet if the 3D mother wavelet satisfies certain conditions. So, the 3D wavelet transform of a 3D image can be implemented by the 2D wavelet transform of its X-ray transform projection and it will contribute to the reduction complexity and computation time during image processing. What is more, it can also avoid noise transfer and amplification during the processing of CT image reconstruction.

## 1. Introduction

Wavelet analysis is developed as a new method for time-frequency analysis in the 1980s. Because it can perform well both on time and frequency domain, we can overcome the disadvantage that cannot be localized on time domain in classical Fourier analysis. Thus, it will be difficult for us to do some practical analyses with Fourier analysis, such as analysis of upheaval signal, determination of trouble point (catastrophe point) timely, and localization of the image edge defects accurately. Wavelet analysis has been a discipline with wide applications since its advent, and significant achievements have been achieved in some of the applications in image compression, edge detection, image denoising, image fusion, nondestructive testing, watermark, finance, military industry, geophysical prospecting, and so forth [1–12]. In recent years, scholars have begun to apply wavelet analysis to image reconstruction from projection data and image processing in Industry Computerized Tomography (ICT) [13–15].

Under the basic theory of image processing, if we want to get some features such as image edge or texture feature, certain transformation of the original image is needed for further process in the transform domain to get the desired characterization. We can also understand the process in the sense of filtering; that is, certain filter can be utilized to get image features. While the fact is that the design and implementation of high dimensional filter are difficult and time consuming.

Two-dimensional Radon transform (different angles of line integral) can well reflect the relationship between scanned object density and ray projection in two-dimensional CT. For that reason, nontensor product relationship between 2D mother wavelet function and 1D mother wavelet function from the Radon projection of 2D mother wavelet function is given by Li Zeng, and others. And they indicate that Radon projection is 1D mother wavelet function, while the 2D mother function satisfies some conditions. Thus, the 2D wavelet transform of 2D function can be derived from the 1D wavelet transform of 1D projection. This is easy to solve the 1D problem derived from the original 2D problem, which has been concretely applied in ICT [7, 13, 16, 17]. Generally, the actual projection data from CT contain noise more or less, and the noise property of the reconstructed image is nonwhite even if the noise is white [17–20]; then it will be inconvenient for further process of CT image. While the problem could be easier if we process the projection data instead of CT image directly. By this way, computation cost can be greatly reduced and some image processing problems such as feature extraction from CT image are effectively solved.

3D wavelet transforms have been widely used in 3D image compression, edge extraction, noise reduction, and so forth. Compared to the three-dimensional Radon transform (different angles of plane integral), X-ray transform can reflect more realistic relationship between scanning object density and ray projection in three-dimensional CT. In previous papers, we discussed the problem of inverse transformation; namely, the X-ray inverse transformation of 2D mother wavelet is 3D mother wavelet function under certain conditions. So, the 3D mother wavelet is structured by the back projection of 2D mother wavelet functions [21, 22]. In this paper, discussing the problem of normal transformation (the X-ray transformation of 3D mother wavelet is 2D mother wavelet function that satisfy certain conditions) and realizing 3D wavelet transformation through 2D wavelet of the multiangle projection. In the experiments, the projection methods avoid the noise migration and save calculation time in the edge extraction. 

## 2. The *N*-Dimension Wavelet and Wavelet Transform

Suppose *ψ*(**x**) ∈ *L* ^2^(*R* ^*n*^) is a complex-value function (*n* is a positive integer); then *ψ*(**x**) is referred to as mother wavelet function (or wavelet base function) [21, 22] if the following admissibility condition is satisfied:

(1) $$\begin{array}{c} \int_{R^{n}}\frac{{\left|\hat{\psi }\left(w\right)\right|}^{2}}{{\left|w\right|}^{n}}dw=C_{\psi }<+\infty , \end{array}$$

where $\hat{\psi }(w)$ is the Fourier transform of *ψ*(**w**). Assuming *ψ*(**x**) ∈ *L* ^1^(*R* ^*n*^), then $\hat{\psi }(w)$ is continuous, and $\hat{\psi }(w)=0$ according to admissibility condition (1). Consider

(2) $$\begin{array}{c} \hat{\psi }\left(0\right)=\int_{R^{n}}\psi \left(w\right)dw=0. \end{array}$$

Then *ψ*(**x**) is 1D mother wavelet function when *n* = 1, *ψ*(**x**) is 2D mother wavelet function when *n* = 2, and *ψ*(**x**) is 3D mother wavelet function when *n* = 3; we denote them by *ψ*(*x*), *ψ*(*x*, *y*), and *ψ*(*x*, *y*, *z*), respectively. Then *ψ*(**w**) formed a wavelet family functions by scaling and translation as follows:

(3) $$\begin{array}{c} \psi_{a,b}\left(x\right)=a^{-n/2}\psi \left(\frac{x\text{-}b}{a}\right), \end{array}$$

where *a* > 0 is the scaling factor, **b** ∈ *R* ^*n*^ is the translation parameter. *f*(**x**) ∈ *L* ^2^(*R* ^*n*^) is an arbitrary function, and the continuous wavelet transform of *f*(**x**) is defined as follows:

(4) $$\begin{array}{c} W_{f}\left(a,b\right)=\frac{1}{\sqrt{C_{\psi }}}\int_{R^{n}}f\left(x\right){\overset{-}{\psi }}_{a,b}\left(x\right)dx, \end{array}$$

where ${\overset{-}{\psi }}_{a,b}(x)$ is the conjugate function of *ψ*_*a*,**b**_(**x**). Wavelet transform is reversible, and its inversion formula is

(5) $$\begin{array}{c} f\left(x\right)=\frac{1}{\sqrt{C_{\psi }}}\int_{0}^{+\infty }\int_{R^{n}}W_{f}\left(a,b\right)\psi_{a,b}\left(x\right)\frac{da\;db}{a^{n+1}}. \end{array}$$

The 3D wavelet transform of 3D function *f*(*x*, *y*, *z*) is abbreviated as

(6) $$\begin{array}{c} Wf\left(x\right)=f\left(x,y,z\right)***\psi \left(x,y,z\right), \end{array}$$

where ∗∗∗ is 3D convolution.

## 3. X-Ray Transform Projection of 3D Mother Wavelet Function

**Figure 1:**
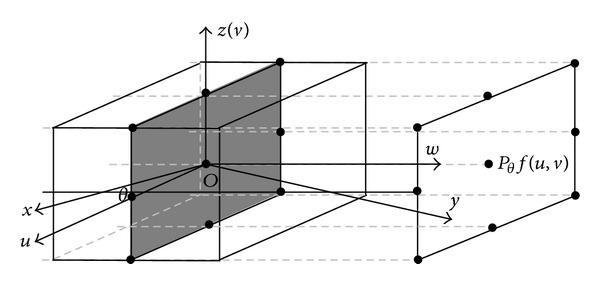
X-ray transform projection.

To simplify our discussion, we introduce the following contents, considering the X-ray transform projection of *f*(**x**) (**x** = (*x*, *y*, *z*) ∈ *R* ^3^). The fixed system of rectangular coordinate (*x*, *y*, *z*) is established with the center of *f*(*x*, *y*, *z*) used as the origin. And we establish a rotating coordinate (*u*, *w*, *v*) by rotating the coordinate system (*x*, *y*, *z*) around the *z*-axis anticlockwise, as shown in Figure 1. Let **u**, **w**, and **v** be unit vectors paralleling to *u*, *w*, and *v* axes respectively, so the following relationship is set up when the rotation angle is *θ*:

(7) $$\begin{array}{c} u=\left(\cos\left(\theta \right),\sin\left(\theta \right),0\right),\\ w=\left(-\sin\left(\theta \right),\cos\left(\theta \right),0\right),\\ v=\left(0,0,1\right). \end{array}$$

Considering the X-ray transform projection of 3D function *f*(*x*, *y*, *z*) along *w*-axis, then the direction of projection is uniquely determined by *θ*. For simplicity and convenience, the direction of projection will be referred to as direction *θ* in the following content. The X-ray transform projection *P*_*θ*_ *f*(*u*, *v*) of 3D function *f*(*x*, *y*, *z*) along direction *θ* is defined as [23, 24]

(8) Pθf(u,v)=∫Rnf(x)δ(x·x−u)δ(x·v−v)dx=∫Rf(uu+vv+tw)dt,

where · is inner product in *R* ^3^.

In 3D CT, the X-ray transform projection can be obtained through collecting the projection data of X-ray scan object. Generally, the X-ray transform projection can be obtained through calculating the integral of 3D image when some image processing tasks are implemented.

**Lemma 1 (3D Fourier Slice Theorem [25])**

(9) $$\begin{array}{c} F_{2}P_{\theta }f\left(\omega_{u},\omega_{v}\right)=F_{3}f\left(\omega_{u}u+\omega_{v}v\right), \end{array}$$

where *F*_*i*_ (*i* = 1,2, 3) denote *i* dimension Fourier transform.

The Fourier slice theorem in 3D can be interpreted as follows. The 2D Fourier transform of the X-ray transform projection of *f*(**x**) along the direction *θ* is equal to the slice plane through the origin of the 3D Fourier transform and with its normal direction parallel to *θ* (as shown in Figure 1). It plays an important role in connecting the X-ray transform projection of *f*(**x**) and its Fourier transform. When the case is in 2D, X-ray transform is Radon transform, and readers can find more details about Radon transform in the pieces of literature [26–28].

**Lemma 2**

Fourier transform is a one-to-one mapping on *L* ^2^(*R*) [25]. This lemma could be generalized to high dimensional situations, and the following formula is established for arbitrary *f*(**x**) ∈ *L* ^2^(*R* ^*n*^):

(10) $$\begin{array}{c} {\left(\int_{R^{n}}{\left|f\left(x\right)\right|}^{2}dx\right)}^{1/2}={\left(\frac{1}{2\pi }\right)}^{n/2}{\left(\int_{R^{n}}{\left|\hat{f}\left(w\right)\right|}^{2}dw\right)}^{1/2}. \end{array}$$

## 4. The Relationship 3D Wavelet and 2D Wavelet

The section is the core content of this paper. The X-ray transformation of 3D mother wavelet is 2D mother wavelet function that satisfy certain conditions, and 3D wavelet transformation is realized by 2D wavelet of the multiangle projection.

**Theorem 3**

If *ψ*(*x*, *y*, *z*) ∈ *L* ^2^(*R* ^3^), with its Fourier transform $\hat{\psi }(\omega_{x},\omega_{y},\omega_{z})$, then *P*_*θ*_ *ψ*(*u*, *v*) ∈ *L* ^2^(*R* ^2^) if the following condition is satisfied:

(11) $$\begin{array}{c} \underset{\theta ,\left|\omega_{u}\right|\leq 1}{\max}\int_{-\infty }^{+\infty }{\left|F_{2}P_{\theta }\psi \left(\omega_{u},\omega_{v}\right)\right|}^{2}d\omega_{v}=k<+\infty , \end{array}$$

where *P*_*θ*_ *ψ*(*u*, *v*) is the X-ray transform of *ψ*(*x*, *y*, *z*) along direction *θ*, its Fourier transform is *F*_2_ *P*_*θ*_ *ψ*(*ω*_*u*_, *ω*_*v*_), and *k* is a certain constant.

**Proof**

Since *ψ*(*x*, *y*, *z*) ∈ *L* ^2^(*R* ^3^), according to Lemma 2, we obtain

(12) $$\begin{array}{c} {∭}_{-\infty }^{+\infty }{\left|\hat{\psi }\left(\omega_{x},\omega_{y},\omega_{z}\right)\right|}^{2}d\omega_{x}\;d\omega_{y}\;d\omega_{z}<+\infty . \end{array}$$

Implement the transform of cylindrical coordinate

(13) ωx=ωucos⁡θ,ωy=ωusinθ, (0≤θ≤2π)ωz=ωv.

Then we could obtain

(14) $$\begin{array}{c} \int_{0}^{2\pi }\int_{-\infty }^{+\infty }\int_{-\infty }^{+\infty }{\left|\hat{\psi }\left(\omega_{u}u+\omega_{v}v\right)\right|}^{2}\left|\omega_{u}\right|d\omega_{u}\;d\omega_{v}\;d\theta <+\infty , \end{array}$$

with $|\hat{\psi }(\omega_{u}u+\omega_{v}v){|}^{2}|\omega_{u}|\geq 0$. Then by utilizing Lemma 1, we obtain

(15) $$\begin{array}{c} \iint_{-\infty }^{+\infty }{\left|F_{2}P_{\theta }\psi \left(\omega_{u},\omega_{v}\right)\right|}^{2}\left|\omega_{u}\right|d\omega_{u}\;d\omega_{v}<+\infty ; \end{array}$$

then

(16) ∬−∞+∞|F2Pθψ(ωu,ωv)|2dωu dωv  ≤∫−1+1dωu∫−∞+∞|F2Pθψ(ωu,ωv)|2dωv   +∫−∞−1dωu∫−∞+∞|F2Pθψ(ωu,ωv)|2|ωu|dωv   +∫1+∞dωu∫−∞+∞|F2Pθψ(ωu,ωv)|2|ωu|dωv,

while

(17) $$\begin{array}{c} \int_{-1}^{1}d\omega_{u}\int_{-\infty }^{+\infty }{\left|F_{2}P_{\theta }\psi \left(\omega_{u},\omega_{v}\right)\right|}^{2}d\omega_{v}\leq 2k<+\infty , \end{array}$$

Hence,

(18) $$\begin{array}{c} \iint_{-\infty }^{+\infty }{\left|F_{2}P_{\theta }\psi \left(\omega_{u},\omega_{v}\right)\right|}^{2}d\omega_{u}\;d\omega_{v}<+\infty . \end{array}$$

Consequently, we obtain *P*_*θ*_ *ψ*(*u*, *v*) ∈ *L* ^2^(*R* ^2^) by again referring to Lemma 2.

**Theorem 4**

Suppose that the X-ray transform of 3D mother wavelet function *ψ*(*x*, *y*, *z*) along direction *θ* is *P*_*θ*_ *ψ*(*u*, *v*); then *P*_*θ*_ *ψ*(*u*, *v*) is 2D mother wavelet function if the following conditions are satisfied.

(1) The Fourier transform *F*_2_ *P*_*θ*_ *ψ*(*ω*_*u*_, *ω*_*v*_) of *P*_*θ*_ *ψ*(*u*, *v*) satisfies the admissibility condition
  (19) $$\begin{array}{c} \iint_{-\infty }^{+\infty }{\left|F_{2}P_{\theta }\psi \left(\omega_{u},\omega_{v}\right)\right|}^{2}{\left(\omega_{u}^{2}+\omega_{v}^{2}\right)}^{-1}d\omega_{u}\;d\omega_{v}<+\infty . \end{array}$$
(2) max⁡_*θ*,|*ω*_*u*_|≤1_ | *F*_2_ *P*_*θ*_ *ψ*(*ω*_*u*_, *ω*_*v*_)|^2^ *dω*_*v*_ = *k* < +*∞* (*k* is a certain constant).

**Proof**

*ψ*(*x*, *y*, *z*) is 3D mother wavelet as aforementioned; then *ψ*(*x*, *y*, *z*) ∈ *L* ^2^(*R* ^3^). Then *P*_*θ*_ *ψ*(*u*, *v*) ∈ *L* ^2^(*R* ^2^) according to Theorem 3. Because *P*_*θ*_ *ψ*(*u*, *v*) satisfies admissibility condition, *P*_*θ*_ *ψ*(*u*, *v*) is a 2D mother wavelet function.

Mother wavelet function has localization properties on time-frequency domain, so the condition of Theorem 4 is satisfied easily.

**Example 5**

Take the first derivative of 3D Gaussian function

(20) $$\begin{array}{c} g\left(x,y,z\right)=\exp\left(-\frac{\left(x^{2}+y^{2}+z^{2}\right)}{2}\right), \end{array}$$

and it is 3D mother wavelet function.

(1) Let *ψ*_1_(*x*, *y*, *z*) = ∂*g*(*x*, *y*, *z*)/∂*x* = −*x*exp⁡−(*x* ^2^ + *y* ^2^ + *z* ^2^)/2); then

(21) ψ^1(ωuu+ωvv)=jωuexp⁡(−(ωu2+ωv2)2)cos⁡λ,(0≤θ<2π);

hence

(22) $$\begin{array}{c} F_{2}P_{\theta }\psi_{1}\left(\omega_{u},\omega_{v}\right)=j\omega_{u}\exp\left(-\frac{\left(\omega_{u}^{2}+\omega_{v}^{2}\right)}{2}\right)\cos\theta . \end{array}$$

Next, we will verify that *P*_*θ*_ *ψ*_1_(*u*, *v*) satisfies the conditions of Theorem 4.

Firstly,

(23) ∬−∞+∞|F2Pθψ1(ωu,ωv)|2(ωu2+ωv2)−1dωu dωv  =∬−∞+∞ωu2exp⁡(−(ωu2+ωv2)2)(cos⁡θ)2   ×(ωu2+ωv2)−1dωu dωv  =12π32cos⁡2θ<+∞;

that is, *P*_*θ*_ *ψ*_1_(*u*, *v*) satisfies admissibility conditions.

Secondly, for ∀*θ* ∈ [0,2*π*) and *w*_*u*_ ∈ [−1,1],

(24) ∫−∞+∞|F2Pθψ1(ωu,ωv)|2dωv  =∫−∞+∞ωu2exp⁡(−(ωu2+ωv2)2)(cos⁡θ)2dωv  =π2wu2e−wu2(cos⁡θ)2≤π2=k<+∞.

So, *P*_*θ*_ *ψ*_1_(*u*, *v*) satisfies the conditions of Theorem 4.

Take 2D Fourier inverse transform of *F*_2_ *P*_*θ*_ *ψ*_1_(*u*, *v*); we can obtain

(25) Pθ1ψ(u,v)=∂g1(u,v)∂ucos⁡θ=−uexp⁡(−(u2+v2)2)cos⁡θ,(0≤θ<2π),

where *g*_1_(*u*, *v*) = exp⁡(−(*u* ^2^ + *v* ^2^)/2) is 2D Gaussian function. Thus, the X-ray transform projection of *ψ*_1_(*x*, *y*, *z*) is

(26) $$\begin{array}{c} P_{\theta }^{1}\psi \left(u,v\right)=-u\exp\left(-\frac{\left(u^{2}+v^{2}\right)}{2}\right)\cos\theta ,\;\left(0\leq \theta <2\pi \right), \end{array}$$

which is 2D mother wavelet function.

(2) Similarly, let

(27) $$\begin{array}{c} \psi_{2}\left(x,y,z\right)=\frac{\partial g\left(x,y,z\right)}{\partial y}=-y\exp\left(-\frac{\left(x^{2}+y^{2}+z^{2}\right)}{2}\right). \end{array}$$

The X-ray transform projection of *ψ*_2_(*x*, *y*, *z*) is

(28) $$\begin{array}{c} P_{\theta }^{2}\psi \left(u,v\right)=-u\exp\left(-\frac{\left(u^{2}+v^{2}\right)}{2}\right)\sin\theta ,\;\left(0\leq \theta <2\pi \right), \end{array}$$

which is 2D mother wavelet function where *ψ*_1_(*x*, *y*, *z*) and *ψ*_2_(*x*, *y*, *z*) are the first derivatives of smooth Gaussian function *g*(*x*, *y*, *z*) respectively.

While the module of wavelet transform of the image can obtain maximal value, which could be used for three-dimensional edge detection (*ψ*_1_(*x*, *y*, *z*) and *ψ*_2_(*x*, *y*, *z*) are mother wavelet functions). What's more, its X-ray transform projections are also mother wavelet functions and the first derivative of 2D smooth functions (including direction factor), which can be used to detect the catastrophe point of a 2D signal (especially for the grayscale catastrophe point with the singularity direction, such as the edge point of an image). The proof of the X-ray transformation of 3D Mexican-hat wavelet is parallel to a 2D mother wavelet function. 

 For compact support 3D mother wavelet, Theorem 6 can be utilized to replace Theorem 3.

**Theorem 6**

If *ψ*(*x*, *y*, *z*) ∈ *L* ^2^(*R* ^3^) is compactly supported, that is

(29) $$\begin{array}{c} \psi \left(x,y,z\right)=0,\;\left(x^{2}+y^{2}+z^{2}>r^{2}\right), \end{array}$$

and *P*_*θ*_ *ψ*(*u*, *v*) is the X-ray transform projection of *ψ*(*x*, *y*, *z*), then *P*_*θ*_ *ψ*(*u*, *v*) ∈ *L* ^2^(*R* ^2^).

**Proof**

Since

(30) ∫−∞+∞[Pθψ(u,v)]2du dv  =∬−∞+∞[∫−∞+∞ψ(uu+vv+tw)dt]2du dv  =∬−∞+∞[∫−r+rψ(uu+vv+tw)dt]2du dv,

according to the Cauchy-Schwarz inequality [29], we have

(31) ∬−∞+∞[∫−r+rψ(uu+vv+tw)dt]2du dv  ≤2r∬−∞+∞∫−r+r[ψ(uu+vv+tw)]2dt du dv  =2r∭−∞+∞[ψ(uu+vv+tw)]2dt du dv  =2r∭−∞+∞[ψ(x,y,z)]2dx dy dz<+∞.

Consequently, *P*_*θ*_ *ψ*(*u*, *v*) ∈ *L* ^2^(*R* ^2^) is supported.

## 5. 2D Realization of 3D Wavelet Transform

The method of realizing 3D wavelet transformation through the 2D wavelet transformation of the multi-angle X-ray transformation projections is presented in this section. 3D wavelet transforms have wide applications in 3D image processing such as image compression, edge extraction, denoising, and so forth. While the design and realization of high dimensional filters are difficult and time consuming. For CT images, noise is included in actual projection data, and the postprocessing for CT images will be difficult because the noise of the reconstructed image maybe nonwhite even if the noise is white. The problem will be easily overcome if the projection of 2D replaces the process of 3D image, and the time consumtion will be saved a lot. Then we derive the 2D realization formula of 3D wavelet transform as follows.

Suppose *f*(*x*, *y*, *z*) and *h*(*x*, *y*, *z*) are 3D functions. Let *s*(*x*, *y*, *z*) = *f*(*x*, *y*, *z*)∗∗∗*h*(*x*, *y*, *z*); their X-ray transform projections along direction *θ* (0 ≤ *θ* < 2*π*) are denoted by *P*_*θ*_ *f*(*u*, *v*), *P*_*θ*_ *h*(*u*, *v*), and *P*_*θ*_ *s*(*u*, *v*), respectively, and their 3D Fourier transforms are denoted by *F*(*ω*_*x*_, *ω*_*y*_, *ω*_*z*_), *H*(*ω*_*x*_, *ω*_*y*_, *ω*_*z*_), and *S*(*ω*_*x*_, *ω*_*y*_, *ω*_*z*_), respectively.

On the one hand, according the Fourier slice theorem of Lemma 1 and the properties of Fourier transform, the X-ray transform of these two functions convolution along direction *θ* could be expressed as

(32) Pθ(f(x,y,z)∗∗∗h(x,y,z))  =F2−1(S(ωuu+ωvv))  =F2−1(F(ωuu+ωvv)H(ωuu+ωvv)).

On the other hand, the convolution of X-ray transforms of these two functions along direction *θ* could be expressed as

(33) Pθf(x,y,z)∗∗Pθh(x,y,z)  =F2−1(F2(Pθf(x,y,z)∗∗Pθh(x,y,z)))  =F2−1(F2(Pθf(x,y,z))F2(Pθh(x,y,z)))  =F2−1(F(ωuu+ωvv)H(ωuu+ωvv)),

where *F*_2_^−1^ is 2D inverse Fourier transform. Combined with formulas (32) and (33), we obtain

(34) Pθ(f(x,y,z)∗∗∗h(x,y,z))  =Pθf(x,y,z)∗∗Pθh(x,y,z).

That is, the X-ray transform of the convolution of two 3D functions is equal to the convolution of the X-ray transform of two functions, whose property is called the distributive convolution of X-ray transform, where ∗∗∗ and ∗∗ are expressed as the convolution of *R* ^3^ and *R* ^2^, respectively.

When *ψ*(*x*, *y*, *z*) satisfies the condition of Theorems 3 and 4, X-ray transform projection *P*_*θ*_ *ψ*(*u*, *v*) along direction *θ* (0 ≤ *θ* < 2*π*) is 2D mother wavelet function. According to the property of the distributive convolution of X-ray transform, the X-ray transform projection of 3D wavelet transform of 3D function *f*(*x*, *y*, *z*) (*Wf*(*x*, *y*, *z*) = *f*(*x*, *y*, *z*)∗∗∗*ψ*(*x*, *y*, *z*)) along direction *θ* (0 ≤ *θ* < 2*π*) is expressed as

(35) $$\begin{array}{c} P_{\theta }Wf\left(u,v\right)=P_{\theta }f\left(u,v\right)**P_{\theta }\psi \left(u,v\right). \end{array}$$

In fact, it can be interpreted as the 2D wavelet transform of X-ray transform projection *P*_*θ*_ *f*(*u*, *v*) of 3D function *f*(*x*, *y*, *z*). Then 3D wavelet transform will be available by taking the X-ray inverse transform of *P*_*θ*_ *Wf*(*u*, *v*) (0 ≤ *θ* < 2*π*). 

## 6. Experiments and Discussion

**Figure 2:**
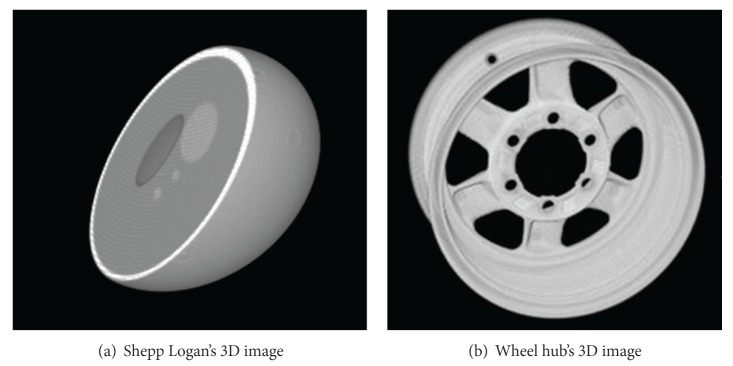
3D Shepp Logan and wheel hub images.

**Figure 3:**
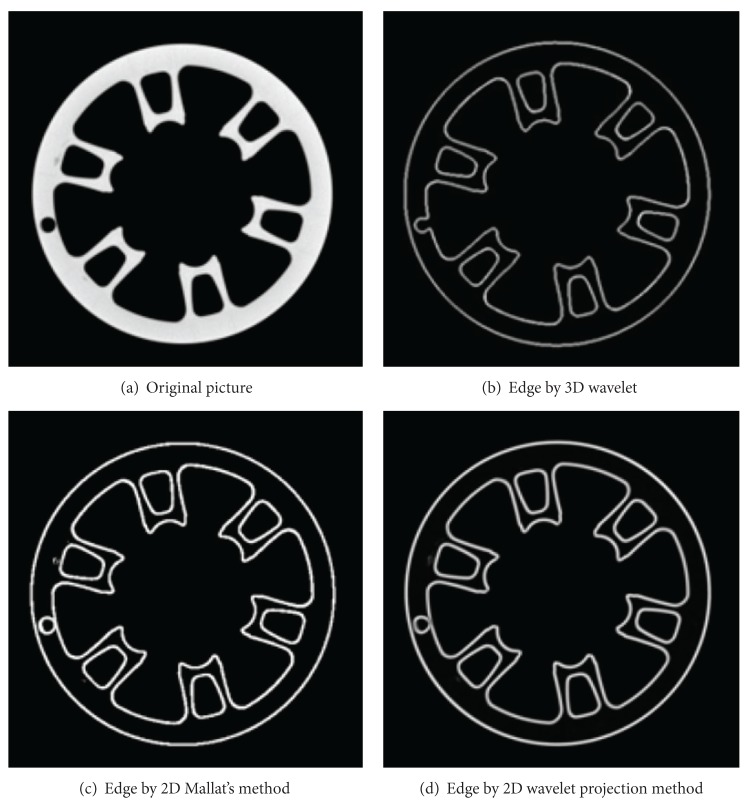
One of 75 wheel hub's CT slices and its edge picture.

**Figure 4:**
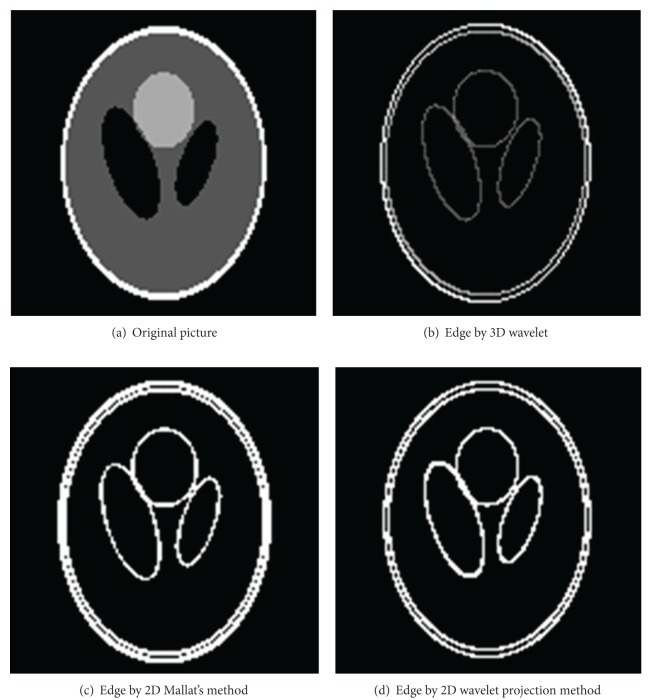
One of 128 Shepp Logan's CT slices and its edge picture.

For practical validation, a series of experiments was performed on various images including the sequence of 75 wheel hub's CT slices [30] (one slice of them is shown in a following paper) and sequence of 128 Shepp Logan's CT slices. 3D wavelet transformation method extracted the edge of 3D CT volume data directly, while their X-ray transformation projections were used to extract the edge of 2D slice. In contrast, we used Mallat's wavelet method extract edges. We show the 3D mapping of wheel hub and Shepp Logan in Figure 2. Mallat's wavelet method, 2D X-ray transformation projection method, and 3D wavelet method are used to extract the edges, with results shown in Figures 3 and 4. From the edge pictures on visual inspection, the experimental results are close to 3D wavelet method and 2D wavelet method (Mallat's wavelet method, 2D wavelet projection method), but 3D wavelet method costs more computation time than 2D wavelet method.

The computational complexity of 3D wavelet method is *O*(*S* × *N* ^2^ × *M* ^3^), however; the complexity of 2D X-ray transformation projection method is *O*(*N* ^2^ × *M* × (*M* + 1)), where *S* represents the number of CT slices, *N* is the size of the image, and *M* is the size of the wavelet; *M* is smaller than *N*. The computational cost of the edge detection methods is shown in Table 1. The data in Table 1 shows that 3D wavelet method costs somewhat more computation time than Mallat's method and 2D X-ray transformation projection method. This paper compares the efficiency of three algorithms. It can be concluded from the following result that the algorithm's efficiency increases greatly.

In short, comparing the method of 3D wavelet transform with 2D wavelet; retaining the similar detail information of binary edges. The computational cost of 2D wavelet is shorter compared to the projection method, 2D Mallat's wavelet method consumed computation time is shorter, but the result of extracted edge is affected by the value of threshold (the extracted edge is rough under the low threshold, on the contrary, the edge fractured easily). But the 2D projection wavelet extracted edge is more finer and continuous.

**Figure 5:**
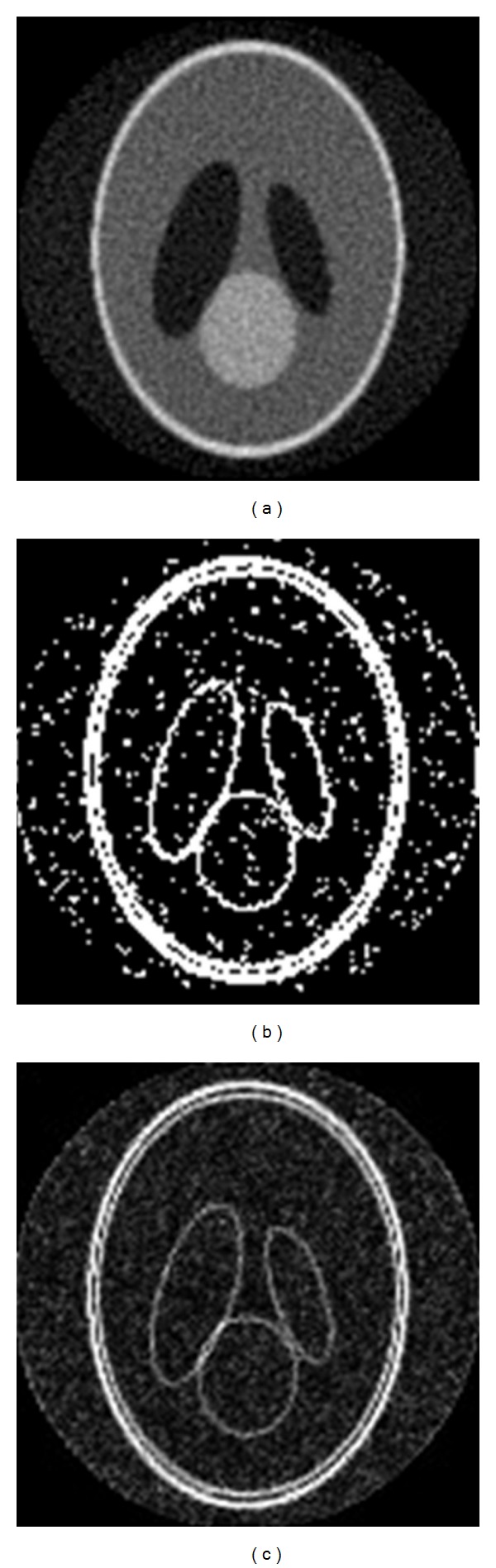
Shepp Logan's reconstructed image and its edge picture.

And we discuss the CT projection data and the CT image, respectively, extracted the edge of Shepp Logan's data (contain gauss noise with mean zero and variance 0.1) as shown in Figure 5.  Figure 5(a) is the reconstructed picture; Figure 5(b) is directly extracted edge of the reconstructed picture, and Figure 5(c) is extracted edge of the projection data. The experimental result demonstrates that the method of 2D projection wavelet extracted edge has better results.

From these pictures, it can be concluded from Figure 5 that 3D wavelet transform of the reconstructed image can be converted to 2D wavelet transform of its projection data, which would avoid the transfer and amplification of the noise during the reconstruction procedure. 

## 7. Conclusion

 In this paper, the nonetensor relationship between 3D mother wavelet function and 2D mother wavelet function is derived from the X-ray transform projection of a 3D mother wavelet function, and X-ray transform projection is 2D mother wavelet function when 3D mother wavelet function satisfies certain conditions. Consequently, 3D wavelet transform of 3D image can be realized by the 2D wavelet transform of X-ray transform projection. The conclusion in this paper can be widely applied to 3D wavelet transform (such as 3D image noise reduction by filtering, edge feature extracting, etc.). The method can also be applied to computerized tomography (CT), and the process towards 3D wavelet transform of the reconstructed image can be converted to the other process towards 2D wavelet transform of its projection data, which will avoid the transfer and amplification of the noise during the reconstruction procedure. And our major research work will focus on the applications of the algorithm in CT industry. What is more, 3D wavelet transform (and its mother wavelet) and 2D wavelet transform (and its mother wavelet) are linked by nontensor product, which is also significant in theory.

## Figures and Tables

**Table 1:** The computational cost of the three methods (wheel hub).

Method	3D wavelet	Mallat's wavelet	2D X-ray transformation projection

Computational cost (s)	1.64	0.43	0.74

Note: computational cost is measured in seconds for processing a 452 × 452 image. Every data in the table above is the average of all slice experiments.
